# Identification of hidden subtypes in occupational health examinations and their biomedical characteristics using graph-enhanced deep representation learning

**DOI:** 10.3389/fpubh.2026.1847173

**Published:** 2026-07-13

**Authors:** Zhaoli Wang, Xuhui Chen, Wei Xiang, Ying Wang, Yang Sun, Yan Pan, Chunhuo Zhang, Yu Ye

**Affiliations:** 1 Department of Labor and Health, China Railway Harbin Group Co., Ltd., Harbin, Heilongjiang, China; 2 Harbin Railway Disease Prevention and Control Center, China Railway Harbin Group Co., Ltd., Harbin, Heilongjiang, China; 3 Heilongjiang Provincial Geriatric Hospital, Harbin, Heilongjiang, China; 4 School of Medicine, University of Electronic Science and Technology of China, Chengdu, China; 5 Heilongjiang Provincial Health Commission, Harbin, Heilongjiang, China

**Keywords:** deep learning, graph neural network, health examination, hidden subtype, occupational health, real-world data, risk stratification

## Abstract

**Purpose:**

Occupational health examinations have long relied on conventional health classes to assess worker health status. However, individuals within the same examination class may differ substantially in risk sources, pathophysiological background, and potential progression patterns. This study aimed to determine whether stable hidden health subtypes exist beyond conventional occupational health classifications in a large real-world cohort and to evaluate the utility of graph-enhanced deep representation learning for subtype discovery and biomedical interpretation.

**Methods:**

We included 63,988 occupational health examination records collected from five organizational units between 2022 and 2025. A standardized master dataset was constructed by harmonizing anthropometric measures, blood pressure, glucose metabolism, lipid metabolism, liver function, kidney function, inflammatory markers, and vascular-related indicators. We first established a benchmark panel including conventional statistical models, tree-based models, and neural network models. We then developed a domain-aware prototype tabular network (DAPTN) and its graph-enhanced variant (Graph-DAPTN) to learn individual-level latent health representations, prototype distances, and hidden subtype structure. The identified subtypes were further interpreted in terms of clinical abnormality burden, risk gradients, temporal drift, and cross-domain stability.

**Results:**

The cohort exhibited substantial heterogeneity across units and years, as well as strong coupling among biomedical indicators. Linear models showed limited performance in capturing complex occupational health phenotypes, whereas nonlinear models performed better overall. In subtype-discovery validation, Graph-DAPTN showed stronger cluster separation and class-subtype concordance than several conventional baselines. Based on this representation, six clinically interpretable hidden subtypes were identified beyond conventional health classes: resilient-healthy, hepato-inflammatory, uric-metabolic, gluco-lipotoxic, hypertensive-homocysteine, and ageing-vascular. These subtypes showed distinct abnormality spectra in glucose and lipid metabolism, blood pressure, liver enzymes, uric acid, homocysteine, and inflammatory burden, and their distributions varied across years and organizational units.

**Conclusion:**

Conventional health classes in occupational health examinations do not fully capture the underlying heterogeneity of worker health status. Graph-enhanced deep representation learning can improve health-state modeling while identifying clinically meaningful hidden subtypes beneath routine classification. These findings may support more refined risk stratification, key-population screening, and unit-level health intervention planning in occupational health practice.

## Graphical abstract

**Figure:**
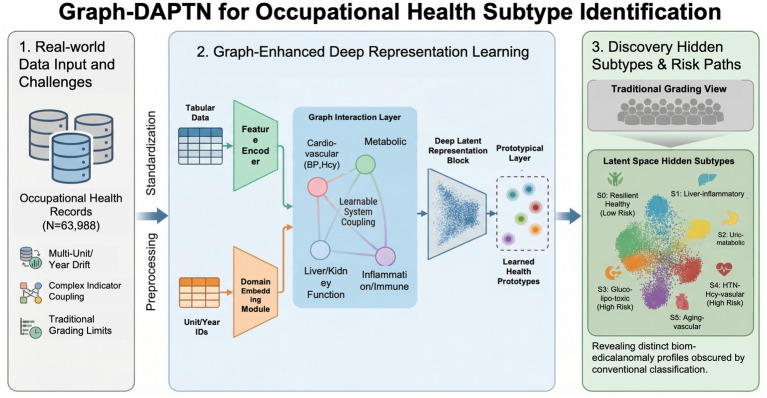

## 1 Introduction

Occupational health examinations play a central role in the early detection and management of health risks among workers (1–3). In routine practice, examination results are commonly summarized into predefined health classes, such as healthy, Class I, Class II, and Class III (4, 5). Although this framework is convenient for administrative management, it may obscure substantial heterogeneity within the same class (6, 7). Workers assigned to the same conventional category may differ in dominant abnormality patterns, underlying biomedical burden, and subsequent management needs (8).

With the accumulation of real-world occupational health data, this hidden heterogeneity has become increasingly important (9). Large-scale examination datasets typically span multiple years, organizational units, and examination settings, and they contain substantial variation in demographic composition, testing completeness, and biomarker distributions (10, 11). In addition, occupational health indicators are strongly coupled across physiological systems, including blood pressure, glucose metabolism, lipid metabolism, liver function, renal function, and inflammatory processes (12, 13). These characteristics make occupational health data both information-rich and methodologically challenging.

Recent advances in artificial intelligence, particularly deep representation learning, provide new opportunities to move beyond conventional label prediction toward latent structure discovery (14, 15). Rather than merely assigning an individual to a predefined examination class, representation learning can capture similarities and differences among individuals in a latent space, making it possible to identify hidden subtypes and characterize their underlying biomedical profiles. For occupational health research, the value of such methods lies not only in predictive performance but also in their ability to reveal clinically meaningful heterogeneity within routine examination categories (16, 17).

However, many previous AI studies in health assessment have focused primarily on predictive accuracy, with limited attention to interpretability, subtype reproducibility, and cross-domain generalization (18). In occupational health settings, these issues are especially important because real-world cohorts are shaped by organizational, temporal, and operational differences. A useful model should therefore not only perform well, but also generate latent structures that remain interpretable and reasonably stable across different years and units (19, 20).

In this study, we aimed to determine whether stable hidden subtypes exist within conventional occupational health classes in a large real-world cohort and whether these subtypes can be linked to interpretable biomedical abnormality profiles. To address this question, we developed and evaluated a graph-enhanced domain-aware prototype tabular network (Graph-DAPTN), and compared it with a broad benchmark panel of conventional machine learning models. We further examined the identified subtypes in terms of clinical abnormality burden, risk gradients, temporal drift, and cross-domain stability.

The primary scientific contribution of this study is not merely improved health-status prediction, but the discovery and conservative characterization of latent occupational-health subtypes beneath routine examination classes. Graph-DAPTN is used as a representation-learning framework to organize multi-system examination indicators into a latent space suitable for subtype discovery, while benchmark prediction, ablation, cluster validation, and cross-domain analyses are used as supporting evidence for the stability and interpretability of the learned representation.

## 2 Materials and methods

### 2.1 Data source and cohort description

This study included 63,988 occupational health examination records collected from five organizational units between 2022 and 2025. All records were derived from routine real-world occupational health examination workflows and reflected the actual operational context of worker health surveillance. The source data covered multiple domains of health assessment, including demographic information, anthropometric indicators, blood pressure, glucose metabolism, lipid metabolism, liver function, kidney function, inflammatory markers, vascular-related indicators, and several auxiliary examination findings.

Because worker identifiers could not be stably and consistently linked across all years, the dataset was analyzed as a repeated cross-sectional occupational health cohort rather than a strict individual-level longitudinal follow-up cohort. Accordingly, the present study focused on latent health structure, hidden subtype discovery, and cross-domain generalization at the population level, rather than formal longitudinal trajectory modeling at the person level.

To preserve the real-world characteristics of occupational health practice, records from different years and units were retained in a unified analytical framework rather than artificially restricted to a homogeneous subset. This design allowed us to explicitly examine temporal drift, organizational heterogeneity, and the stability of learned health representations across multiple operational contexts.

### 2.2 Data cleaning and variable construction

The raw dataset was assembled from multiple Excel workbooks generated in different years and organizational units. Because these source files differed in variable naming conventions, formatting patterns, encoding style, and completeness, we first harmonized all records into a standardized master table and an English variable dictionary. This harmonization step ensured consistent variable semantics across examination batches and made downstream modeling reproducible.

For numeric variables, all values were parsed and cleaned by unifying measurement units, resolving formatting inconsistencies, and converting mixed textual or symbol-containing entries into machine-readable numerical values wherever possible. Obvious format-related anomalies were corrected when they could be safely standardized, while values that could not be reliably interpreted were treated as missing. To preserve clinically informative missingness, missing-indicator variables were generated for major numeric biomarkers and included as additional input features. This allowed the model to distinguish between true measured normality and the absence of a test result.

The core numeric feature set included age, height, weight, body mass index, waist circumference, systolic blood pressure, diastolic blood pressure, heart rate, fasting glucose, glycated hemoglobin, total cholesterol, triglycerides, high-density lipoprotein cholesterol, low-density lipoprotein cholesterol, alanine aminotransferase, aspartate aminotransferase, uric acid, creatinine, blood urea nitrogen, homocysteine, high-sensitivity C-reactive protein, hemoglobin, red blood cell count, and albumin. In addition, several clinically meaningful categorical findings, such as fatty liver status, electrocardiographic findings, chest X-ray findings, major disease history, medical history, and occupational disease information, were retained as structured categorical features.

To better summarize occupational health burden, several derived indicators were also constructed, including metabolic burden, hepatic burden, and composite risk-related variables. These derived variables were not intended to replace the original biomarkers, but to provide additional summaries of multi-indicator abnormality patterns for subtype interpretation and downstream analysis.

For benchmark modeling and deep representation learning, numeric variables were median-imputed and standardized, while categorical variables were encoded into numerical representations after harmonization of category labels. These preprocessing steps were applied consistently within the analytical framework to reduce avoidable technical variation while preserving the clinical structure of the original data.

### 2.3 Benchmark models

To avoid overstating the value of deep learning without sufficient comparison, we established a broad benchmark panel that included linear models, probabilistic models, tree-based models, distance-based models, and neural networks. Specifically, the benchmark set comprised Logistic Regression, Ridge Classifier, Linear SVM, SGD Classifier, Gaussian Naive Bayes, Decision Tree, Random Forest, Extra Trees, AdaBoost, Gradient Boosting, HistGradientBoosting, K-nearest Neighbors, and Multilayer Perceptron.

### 2.4 Graph-DAPTN architecture

Graph-DAPTN was implemented as a graph-enhanced domain-aware prototype tabular network designed for occupational health representation learning. The model was developed to address three major characteristics of occupational health examination data: multi-system biomarker coupling, cross-domain heterogeneity across years and units, and the presence of hidden population structure beneath conventional health classes.

To reflect the clinical organization of occupational health indicators, numeric variables were first partitioned into biomarker groups representing major physiological domains. These groups included anthropometric variables, hemodynamic variables, glycemic variables, lipid variables, hepatic variables, renal variables, hematologic variables, and inflammatory variables. Rather than treating all variables as unrelated scalar inputs, Graph-DAPTN first encoded each biomarker group into a group-level representation. This design allowed the model to capture system-level structure before learning cohort-level latent representations.

A graph-enhanced interaction block was then applied to these group-level tokens. In this block, each biomarker group was projected into a low-dimensional latent token, after which shared contextual information was propagated across groups. This step was intended to model the fact that occupational health burden often arises from interacting physiological systems, such as joint shifts in obesity, glucose, triglycerides, liver enzymes, uric acid, blood pressure, and inflammatory markers. The output of this graph-enhanced block represented a structured summary of inter-system coupling at the individual level.

The graph-enhanced group representation was subsequently fused with three additional information sources: a direct numeric projection of the full continuous input space, categorical embeddings derived from structured examination findings, and domain embeddings representing examination year and organizational unit. The inclusion of domain embeddings allowed the model to explicitly account for real-world variation across time and organizational settings rather than forcing all records into a context-free latent space.

The fused feature representation was then passed through a multilayer latent encoder to produce an individual-level latent health embedding. On top of this embedding, Graph-DAPTN included three complementary output components. First, a supervised classification head was used to predict routine health status labels. Second, an auxiliary reconstruction head was used to reconstruct the original numeric feature space, encouraging the latent representation to preserve clinically relevant information beyond what was needed for label prediction alone. Third, a prototype regularization component was introduced to constrain the embedding space around a set of learned representative health prototypes. This enabled the model to support both classification and subtype-oriented latent organization.

In this framework, each worker’s examination record was not only assigned a predicted health class, but also embedded within a structured latent space that captured proximity to representative health patterns. This design was central to the downstream identification of hidden subtypes and the interpretation of subtype-specific risk structure.

Let xi denote the standardized numeric biomarker vector for record i, ci the encoded categorical examination findings, and di = (yeari, uniti) the domain indicators. Numeric variables were partitioned into G clinically defined biomarker groups, including anthropometric, hemodynamic, glycemic, lipid, hepatic, renal, hematologic, and inflammatory domains. For group g, xig was encoded as hig = phig(xig), where phig is a linear projection followed by a ReLU nonlinearity. The graph-enhanced block used an *a priori* biomarker-system graph rather than an externally estimated patient graph. In implementation, group-level message passing was performed through a uniform contextual interaction, hbar_i = G^−1^ sum_g hig, and htilde_ig = LayerNorm[hig + psi(hbar_i)], where psi is a two-layer message function. This operation is equivalent to propagation over a fully connected group graph with normalized uniform adjacency among biomarker-system tokens.

The propagated group tokens were concatenated with a direct numeric projection, categorical embeddings, and year/unit domain embeddings, and then passed through a multilayer encoder to obtain a latent representation zi. The encoder used hidden layers of 256 and 128 units with batch normalization, ReLU activation, and dropout. The final latent dimension was 24. The training objective combined supervised health-status classification, numeric reconstruction, and prototype regularization:


$$L=L_{\mathrm{CE}}(y,\hat{y})+0.25{\left\|x-\hat{x}\right\|}_{2}^{2}+0.05\;\frac{1}{N}\sum_{i=1}^{N}\min_{m}{\left\|z_{i}-p_{m}\right\|}_{2}$$

Where pm denotes one of 12 learned prototypes. Models were trained with Adam, learning rate 1e-3, batch size 512, and 20 epochs. Hidden subtypes were obtained by applying k-means clustering to zi.

### 2.5 Training details

The latent embedding dimension was set to 24, the prototype count to 12, the batch size to 512, and the learning rate to 1 × 10^−3. The model was trained for 20 epochs using a joint objective consisting of cross-entropy loss for supervised classification, mean squared error for numeric reconstruction, and prototype-distance regularization. Hidden subtype assignments were obtained by applying K-means clustering (*k* = 6) to the learned latent embeddings.

### 2.6 Evaluation strategy

Model robustness was evaluated using random-split validation, leave-one-year-out validation, and leave-one-unit-out validation. Performance was assessed using macro-F1, balanced accuracy, weighted F1, and accuracy. For Graph-DAPTN cross-domain evaluation, each held-out year or unit was tested across multiple random seeds. In addition, ablation experiments were conducted to examine the contributions of the graph-enhanced interaction module, domain embeddings, and prototype regularization.

### 2.7 Subtype baselines and K selection

To directly evaluate hidden subtype discovery, we added unsupervised baselines separate from routine health-status prediction. K-means clustering with *k* = 6 was applied to standardized clinical features, PCA-reduced clinical features, PersonalizedHealthNet latent embeddings, PioneerNet latent embeddings, baseline DAPTN embeddings, and Graph-DAPTN embeddings. A PCA-Gaussian mixture model was included as an additional classical unsupervised clustering baseline. Cluster validity was evaluated using silhouette score, Calinski-Harabasz index, Davies-Bouldin index, repeated-seed ARI/NMI stability, minimum cluster fraction, severe-risk separation, metabolic/hepatic burden separation, and concordance with routine health status.

The number of clusters was evaluated for Graph-DAPTN across *k* = 2 to 10. The final *k* = 6 solution was selected as a compromise between statistical validity, repeated-seed stability, subtype granularity, and biomedical interpretability. We did not treat *k* = 6 as a mathematically unique optimum.

## 3 Results and discussion

### 3.1 The occupational health cohort exhibited large scale, marked heterogeneity, and clear latent structural complexity

**Figure 1:**
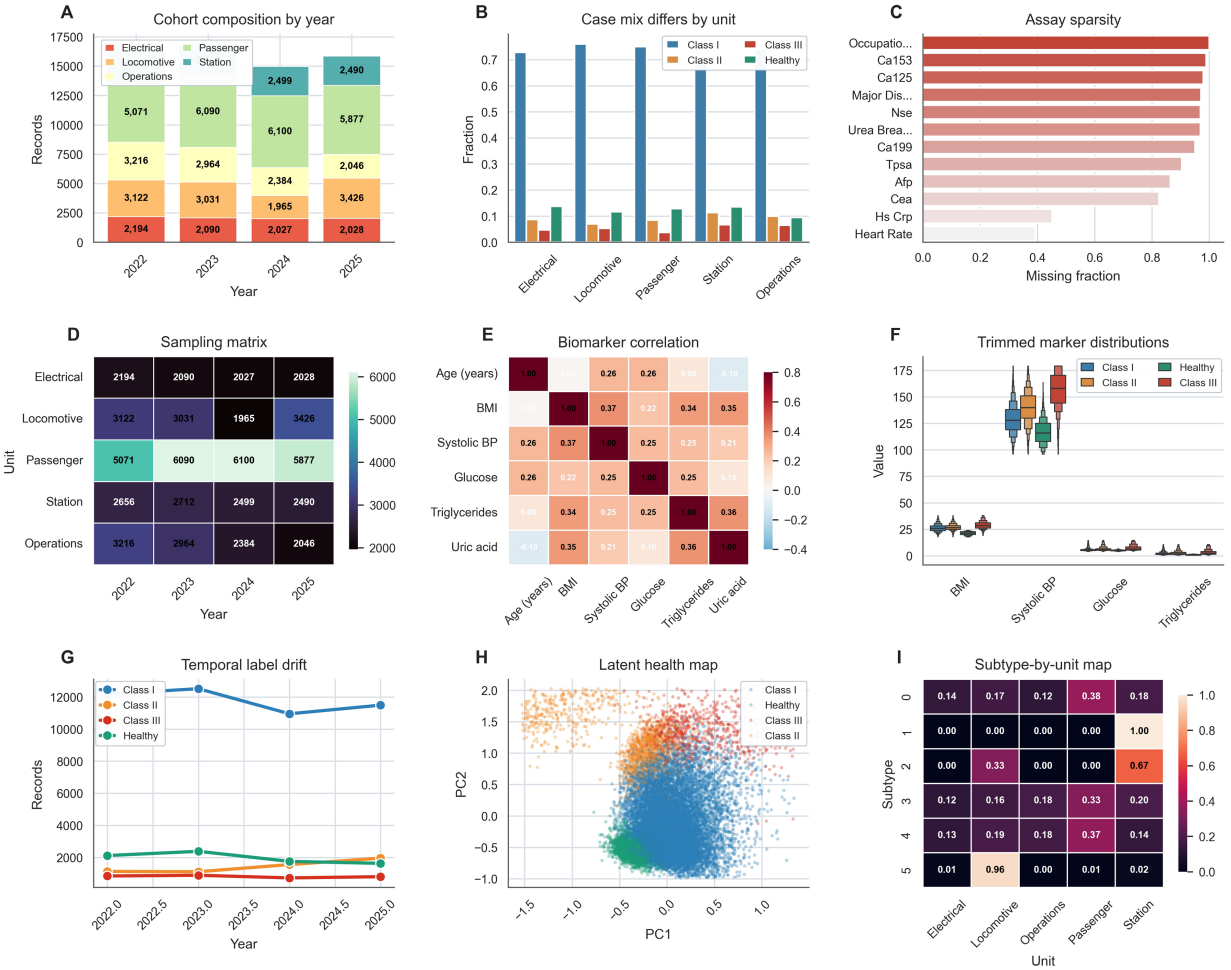
Scale, heterogeneity, and baseline structure of the occupational health cohort. **(A)** Cohort composition by year and organizational unit. **(B)** Case-mix differences across units by conventional health class. **(C)** Assay sparsity of selected variables ranked by missing fraction. **(D)** Sampling matrix across units and years. **(E)** Correlation structure among representative core biomarkers. **(F)** Trimmed distributions of selected markers across conventional health classes. **(G)** Temporal drift in conventional health-label counts. **(H)** Low-dimensional latent health map from the baseline embedding model. **(I)** Subtype-by-unit enrichment map derived from the baseline latent embedding.

The occupational health cohort included 63,988 examination records collected across five organizational units between 2022 and 2025. As shown in Figure 1A, the annual cohort composition also differed by organizational unit, indicating substantial variation in sampling contribution across years. Figure 1B further shows that the case mix of conventional health classes differed across units. In addition, Figure 1C summarizes assay sparsity for selected variables, indicating that the completeness of laboratory and auxiliary examination items was not uniform across the cohort.

This heterogeneity became more evident when the cohort was examined at a finer resolution. Figure 1D shows the sampling matrix across units and years, illustrating that the cohort was structurally unbalanced across organizational and temporal strata. Figure 1E presents the correlation structure among representative core biomarkers, showing that occupational health burden was organized across coupled physiological systems rather than isolated variables. Figure 1F further shows trimmed distributions of selected markers across conventional health classes, indicating that major clinical indicators shifted systematically with routine health status.

From a biomedical perspective, the pattern shown in Figure 1F is especially meaningful. It suggests that the occupational health burden in this cohort was not organized as a series of isolated single-indicator abnormalities, but as coordinated multi-system shifts. For example, obesity, hyperglycaemia, HbA1c elevation, and hypertriglyceridaemia may form one coherent metabolic axis, whereas blood pressure elevation and homocysteine may represent a different vascular-risk-oriented axis. Likewise, liver enzymes and inflammatory markers may define another partially independent route of abnormality. These coupled structures indicate that routine health classes are likely to compress underlying physiological heterogeneity.

This interpretation is supported by the more global structural patterns shown in Figures 1G–I. The temporal label drift in Figure 1G suggests that the cohort changed over time at the level of conventional health labels. The low-dimensional latent health map in Figure 1H indicates that the population may contain nonlinear internal organization beyond routine categories. The subtype-by-unit map in Figure 1I further suggests that latent subgroup structure may also vary across organizational settings.

### 3.2 Graph-DAPTN provides a transparent and robust representation framework for hidden subtype discovery

**Figure 2:**
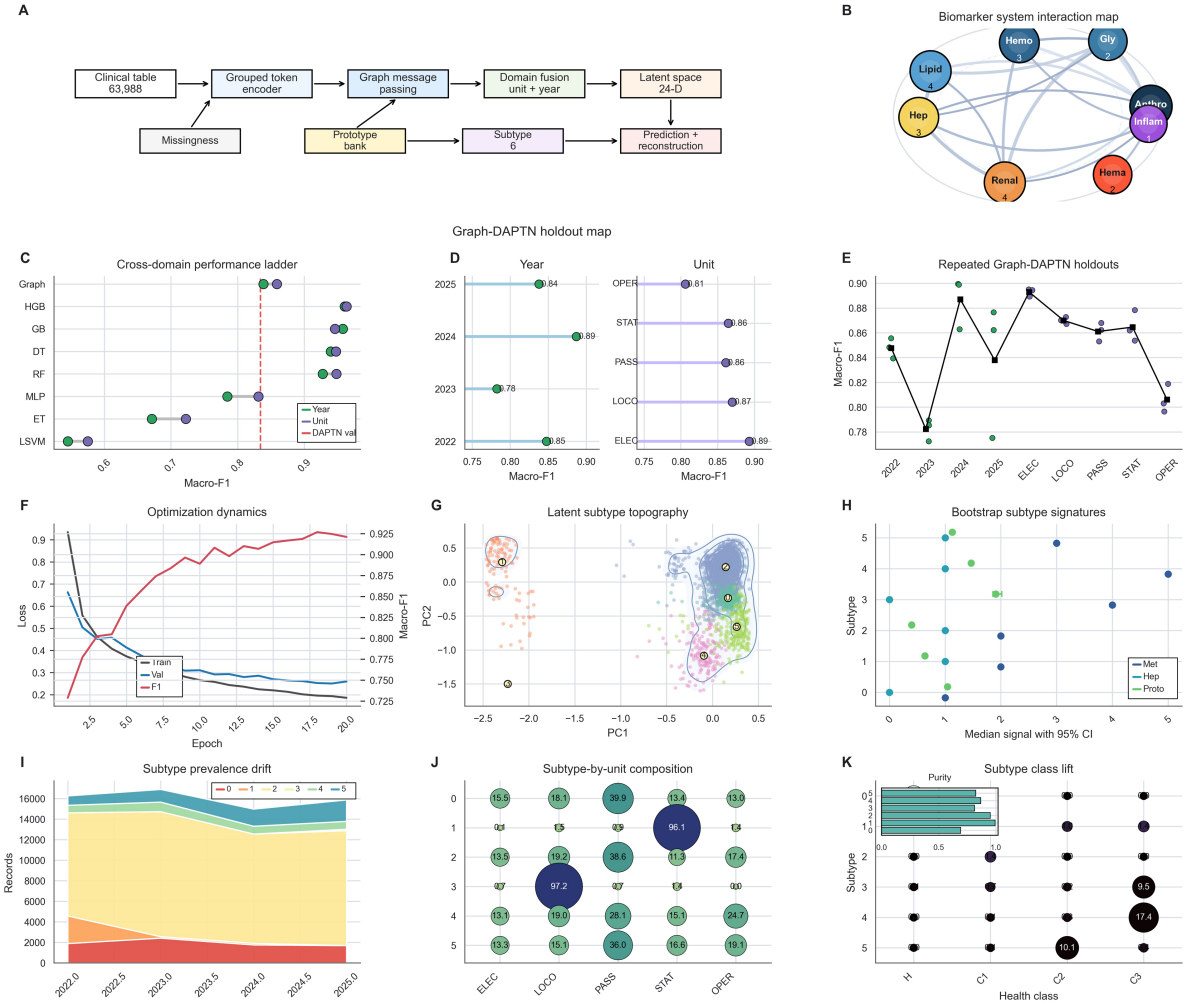
Graph-DAPTN architecture, cross-domain robustness, and latent representation structure. **(A)** Overview of the Graph-DAPTN workflow, including grouped biomarker-token encoding, missingness information, graph message passing, domain fusion by unit and year, prototype-based subtype organization, and prediction/reconstruction objectives. **(B)** Biomarker-system interaction map used to organize examination variables into clinically related systems. Node size indicates the number of variables in each system, and edges indicate graph-based information exchange between systems. **(C)** Cross-domain performance ladder comparing Graph-DAPTN with representative conventional models under year-out and unit-out validation. **(D)** Graph-DAPTN holdout map summarizing mean macro-F1 across individual years and occupational units. **(E)** Repeated Graph-DAPTN holdout results showing run-to-run variability across held-out years and units. **(F)** Optimization dynamics of Graph-DAPTN, including training loss, validation loss, and validation macro-F1 across epochs. **(G)** Latent subtype topography showing the six prototype-defined subtypes in the two-dimensional projection of the learned Graph-DAPTN representation. **(H)** Bootstrap subtype signatures for metabolic-risk score, hepatic-risk score, and prototype distance, shown as median signals with confidence intervals. **(I)** Temporal drift in subtype prevalence across examination years. **(J)** Subtype-by-unit composition showing the distribution of each subtype across occupational units. **(K)** Subtype class lift showing enrichment of latent subtypes within conventional health-status classes.

Graph-DAPTN was designed to move the analysis beyond direct reproduction of conventional occupational health-status labels and toward a structured latent representation suitable for hidden subtype discovery. As shown in Figure 2A, the model transforms the clinical examination table into grouped biomarker tokens, incorporates missingness-aware information, propagates messages through a biomarker-system graph, fuses unit and year domain information, and finally learns a 24-dimensional latent space constrained by prototype and reconstruction objectives. The biomarker-system graph in Figure 2B makes this design more interpretable: anthropometric, glycaemic, lipid, hepatic, renal, haematological, haemodynamic, and inflammatory systems are not treated as isolated variables, but as clinically related modules whose interactions may shape occupational health phenotypes.

The cross-domain evaluation indicates that this representation was not driven only by random train-test splitting. In the performance ladder shown in Figure 2C, Graph-DAPTN remained within the range of strong cross-domain models while explicitly providing a latent subtype structure, rather than only a predicted health-status label. The holdout map in Figure 2D further shows that performance was retained across both calendar years and occupational units, with mean macro-F1 values ranging from 0.782 to 0.887 in leave-one-year-out validation and from 0.806 to 0.893 in leave-one-unit-out validation. The repeated holdout results in Figure 2E show the variability across repeated runs and confirm that the strongest and weakest domains were not hidden by a single average performance value.

The training trajectory provides additional evidence that the model learned a stable representation rather than relying on a degenerate solution. Figure 2F shows a steady decline in training and validation loss, accompanied by an increase in validation macro-F1 that reached approximately 0.90.

Additionally, the latent space learned by Graph-DAPTN showed a structured subtype topography. In Figure 2G, the six prototype-defined subtypes occupy distinguishable regions rather than appearing as arbitrary label overlays. The bootstrap subtype signatures in Figure 2H further suggest that the subtypes differ in their median metabolic-risk, hepatic-risk, and prototype-distance signals, with confidence intervals indicating that these signatures were not dominated by a single unstable sample draw.

Because the data were collected from multiple years and occupational units, we also examined whether the latent subtypes merely reflected sampling domains. Figure 2I shows that subtype prevalence varied over time, which is expected in real-world occupational health surveillance and is consistent with temporal changes in cohort composition. Figure 2J further shows that several subtypes were distributed across multiple units, whereas a small number of rare subtypes showed strong unit enrichment. This pattern suggests that both shared latent health structures and domain-specific phenotypic concentrations were present; therefore, unit and year effects were treated as important robustness factors rather than ignored as nuisance variation.

Figure 2K links the learned subtypes back to conventional health-status labels. Some subtypes were strongly enriched within a single health class, whereas others cut across existing labels, indicating that the model did not simply relabel the original classification. The conventional supervised benchmark against traditional algorithms is retained in Supplementary Appendix A and Supplementary Figure S1.

### 3.3 Graph-DAPTN identifies subtype structure beyond conventional clustering baselines

**Figure 3:**
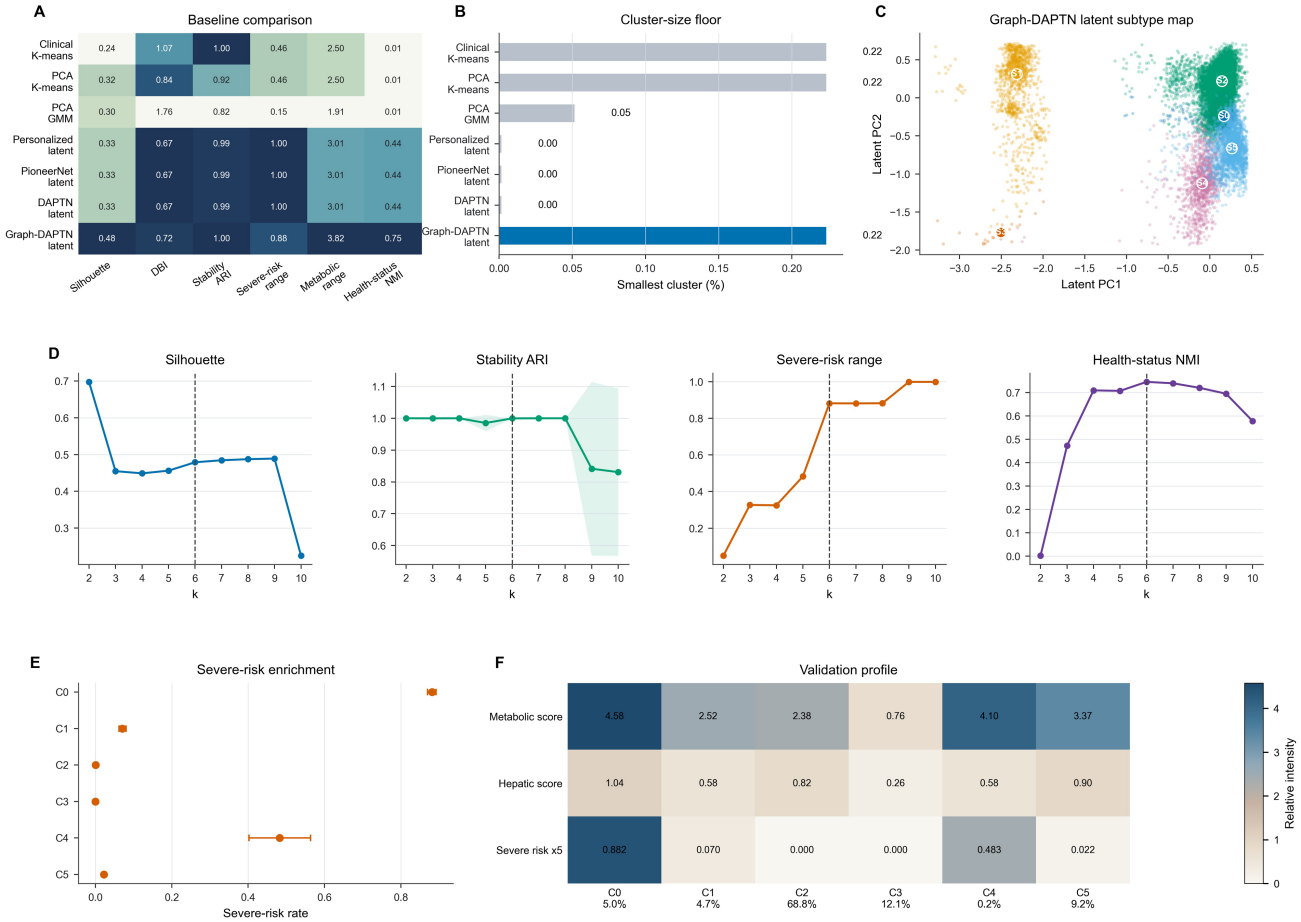
Subtype-discovery validation of the Graph-DAPTN latent representation. **(A)** Comparison of subtype-discovery baselines, including clustering on clinical features, PCA representations, Gaussian mixture modeling, non-graph latent representations, and Graph-DAPTN latent embeddings. Values in the heatmap show raw metrics, while color intensity indicates within-metric scaled performance. **(B)** Smallest-cluster fraction for each baseline, used to evaluate whether a method produced extremely small or unstable clusters. **(C)** Two-dimensional projection of the Graph-DAPTN latent representation showing the prototype-defined subtypes used for subsequent interpretation. **(D)** Sensitivity analysis across *k* = 2 to *k* = 10, including silhouette score, bootstrap stability measured by adjusted Rand index, severe-risk separation, and health-status normalized mutual information. The dashed line indicates *k* = 6. **(E)** Severe-risk enrichment across the six validation clusters. **(F)** Biomedical profile of validation clusters based on metabolic-risk score, hepatic-risk score, and severe-risk intensity. The validation clusters in panels **E–F** are denoted C0-C5 to distinguish them from the final named subtypes S0-S5 shown in panel **C** and subsequent figures.

To address whether the identified subtypes were specific to Graph-DAPTN rather than a generic result of applying K-means to any representation, we performed a subtype-discovery-specific benchmark. Figure 3A compares clustering on clinical features, PCA representations, Gaussian mixture modeling, non-graph latent representations, and Graph-DAPTN latent embeddings. Graph-DAPTN achieved the highest silhouette value among the evaluated representations (0.479) and the strongest alignment with conventional health-status structure (NMI = 0.746), while also preserving high bootstrap stability (ARI = 0.9997). These results do not imply that Graph-DAPTN is always superior for every prediction task; rather, they show that its latent representation provides a more suitable structure for subtype discovery than direct clustering of the raw clinical table or PCA-reduced features.

Cluster-size balance was also considered because a high clustering metric can be misleading if the solution is dominated by extremely small or uninterpretable clusters. Figure 3B shows that Graph-DAPTN did not avoid small clinically important clusters, but its smallest cluster size was comparable to the clinical-feature and PCA K-means baselines. This result is important because the aim was not to force evenly sized groups, but to identify latent phenotypes that are both statistically detectable and clinically interpretable. The latent projection in Figure 3C further illustrates that the learned Graph-DAPTN representation formed structured regions corresponding to the prototype-defined subtypes, rather than appearing as a diffuse label overlay.

We also performed a sensitivity analysis across *k* = 2 to *k* = 10 to ensure that the selected subtype structure was not an arbitrary consequence of fixing the cluster number. As summarized in Figure 3D, *k* = 6 provided a practical balance between internal separation, bootstrap stability, severe-risk separation, health-status alignment, and interpretability; lower k values produced overly coarse partitions, whereas higher k values added fragmentation without improving biological clarity.

The clinical relevance of the resulting validation clusters was supported by risk enrichment and biomedical profile analyses. Figure 3E shows that the validation clusters differed substantially in severe-risk rate, with one cluster showing marked severe-risk enrichment and others showing low or intermediate risk. Figure 3F further shows that the same validation clusters differed in metabolic-risk score, hepatic-risk score, and severe-risk intensity.

As shown in Table 1, subtype membership was significantly associated with routine health-status composition (chi-square *p* < 0.001). Metabolic and hepatic burden scores also differed across subtypes by Kruskal-Wallis tests (both *p* < 0.001). These tests support quantitative differences among latent subtypes but do not imply causal mechanisms.

**Table 1:** Statistical tests and confidence intervals of the 5 subtypes.

Subtype	n	Fraction	Severe-risk rate, 95% CI	Metabolic burden	Hepatic burden
C0	3,183	5.0%	88.2% (87.0–89.2%)	4.58	1.04
C1	2,980	4.7%	7.0% (6.2–8.0%)	2.52	0.58
C2	44,022	68.8%	0.0% (0.0–0.1%)	2.38	0.82
C3	7,765	12.1%	0.0% (0.0–0.0%)	0.76	0.26
C4	143	0.2%	48.3% (40.2–56.4%)	4.10	0.58
C5	5,895	9.2%	2.2% (1.9–2.6%)	3.37	0.90

### 3.4 The subtype atlas reveals heterogeneous latent phenotypes beneath conventional health labels

**Figure 4:**
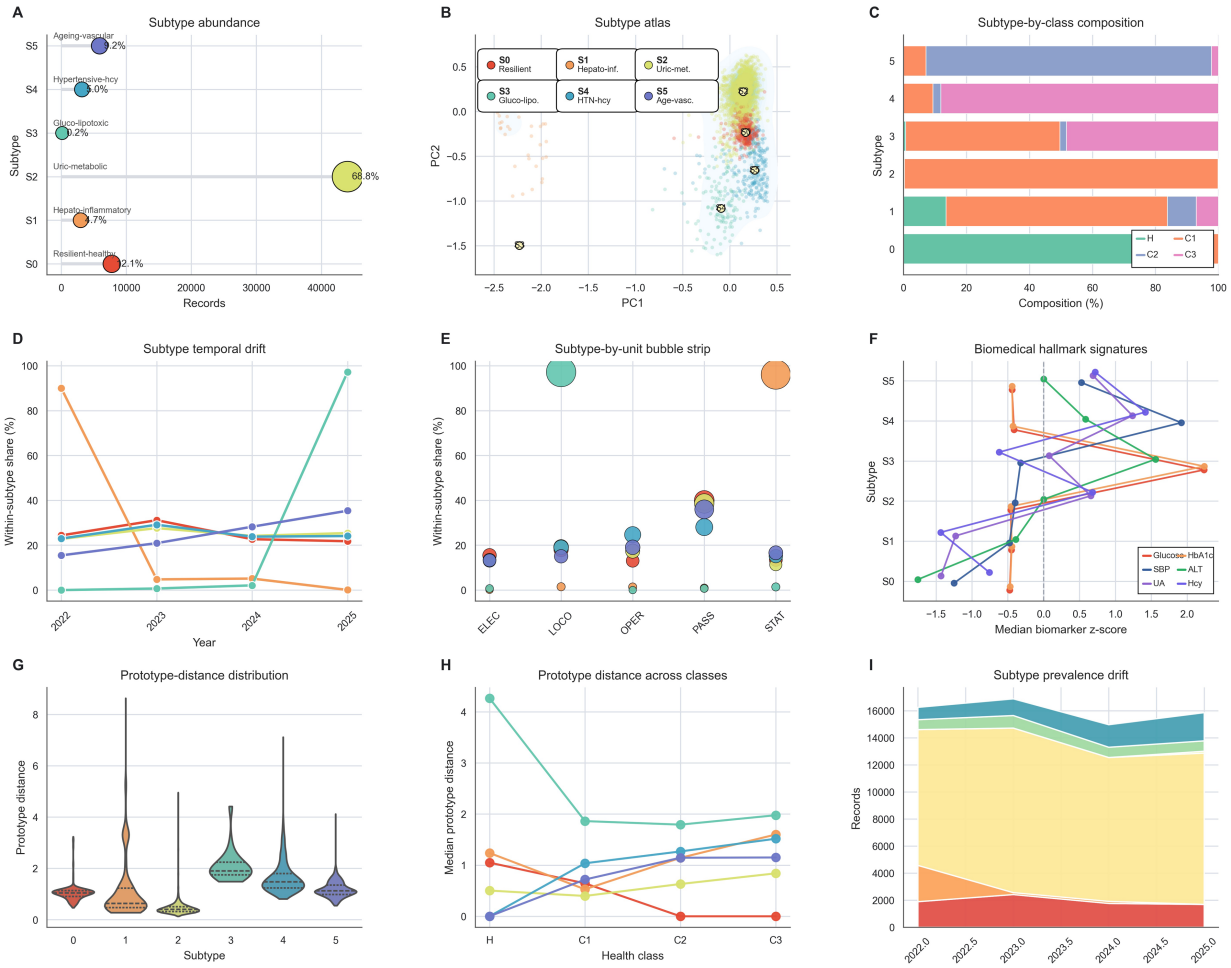
Graph-DAPTN subtype atlas, clinical composition, and domain distribution. **(A)** Abundance of the six named subtypes in the occupational health cohort. **(B)** Latent subtype atlas showing the distribution of named subtypes in the Graph-DAPTN representation space. **(C)** Subtype composition across conventional health-status classes. **(D)** Temporal distribution of each subtype across examination years. **(E)** Unit distribution of each subtype across occupational units. **(F)** Biomedical hallmark signatures of the six named subtypes based on median biomarker z-score deviations. **(G)** Prototype-distance distributions by subtype. **(H)** Prototype-distance shifts across conventional health-status classes. **(I)** Temporal prevalence drift of subtypes.

After validating the latent subtype structure, we examined how the six named subtypes were distributed in the occupational health cohort. Figure 4A shows that the subtype distribution was highly imbalanced: S2 was the largest group (44,022 records; 68.8%), followed by S0 (7,765; 12.1%), S5 (5,895; 9.2%), S4 (3,183; 5.0%), S1 (2,980; 4.7%), and the rare S3 group (143; 0.2%). This imbalance is clinically plausible for a surveillance cohort, because most examinees are expected to fall into broad low-to-moderate-risk phenotypes, whereas rare clusters may represent concentrated abnormal profiles.

The latent subtype atlas in Figure 4B shows that these named subtypes occupied distinguishable regions of the learned representation space. Their relationship with conventional health-status labels was not one-to-one. Figure 4C shows that S0 was predominantly healthy, S2 was almost entirely Class I, S5 was concentrated in Class II, and S4 was concentrated in Class III. In contrast, S1 and S3 showed more mixed class composition, indicating that some latent phenotypes cut across the conventional grading system. This supports the central premise that conventional labels contain hidden phenotypic heterogeneity.

Domain distribution analyses further clarify how the subtypes should be interpreted. Figure 4D shows that most large subtypes were observed across multiple years, whereas S1 and S3 showed strong temporal concentration. Figure 4E shows a similar pattern across units: S0, S2, S4, and S5 were distributed across several units, while S1 was highly enriched in the station unit and S3 in the locomotive unit. These patterns should not be overinterpreted as causal occupational effects; rather, they indicate that latent phenotypes may reflect a mixture of health status, unit composition, examination timing, demographic structure, and work-related context.

**Figure 5:**
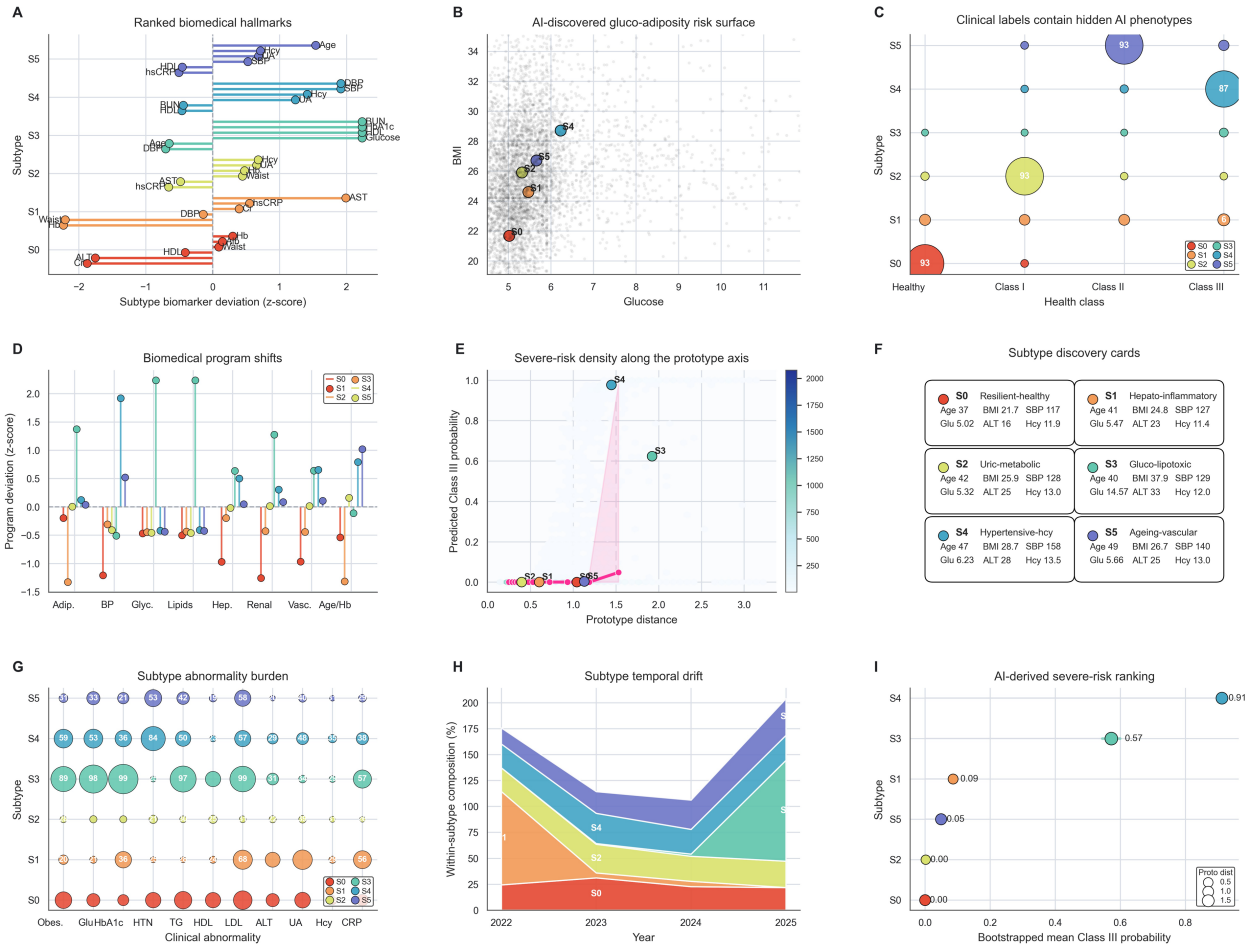
Biomedical characterization and risk interpretation of hidden occupational health subtypes. **(A)** Ranked subtype-specific biomarker deviations, showing the most representative positive and negative biomarkers for each subtype. **(B)** AI-derived gluco-adiposity risk surface showing the relationship among glucose, BMI, and predicted Class III probability, with subtype centers overlaid. **(C)** Distribution of latent subtypes within conventional health-status classes. **(D)** Program-level biomedical shifts across adiposity, blood pressure, glycaemia, lipid, hepatic, renal-purine, vascular, and age-haematologic domains. **(E)** Severe-risk density along the prototype-distance axis. **(F)** Subtype discovery cards summarizing representative clinical values for each subtype. **(G)** Clinical abnormality burden across subtypes based on threshold-defined abnormalities. **(H)** Temporal drift in subtype composition across examination years. **(I)** AI-derived severe-risk ranking based on bootstrapped mean Class III probability.

The biomedical hallmark signatures in Figure 4F provide the first layer of phenotype interpretation. S0 was characterized by generally lower biomarker deviation and was therefore labeled resilient-healthy. S2 showed uric-acid and mild metabolic deviation and was labeled uric-metabolic. S3 showed prominent glucose and HbA1c elevation, supporting its gluco-lipotoxic direction. S4 showed blood-pressure and homocysteine signals and was labeled hypertensive-hcy. S5 showed vascular-risk-related signals and was labeled ageing-vascular after integration with the clinical subtype summaries. S1 showed a relatively stronger hepatic/inflammatory pattern and was labeled hepato-inflammatory. These labels are descriptive phenotype summaries, not causal biological mechanisms, and their expanded biomedical support is examined further in Figure 5.

Prototype-distance analyses support the interpretation that subtypes also differ in their position relative to learned latent prototypes. Figure 4G shows that prototype-distance distributions varied by subtype, and Figure 4H shows that these distances shifted across conventional health classes. Figure 4I further shows that subtype prevalence changed over time, reinforcing that subtype composition is dynamic in a real-world surveillance setting. Overall, Figure 4 translates the statistically validated latent clusters into a clinically interpretable subtype atlas while preserving caution about domain effects and subtype-label interpretation.

### 3.5 Biomedical characterization links hidden subtypes to distinct abnormality burdens and risk pathways

The ranked hallmark analysis in Figure 5A shows that each subtype was defined by a different combination of positive and negative biomarker deviations rather than by a single overall severity scale. This is important because two workers with similar conventional health-status labels may still differ substantially in their metabolic, hepatic, vascular, renal, inflammatory, or age-related abnormality patterns.

The gluco-adiposity risk surface in Figure 5B illustrates one example of this multidimensional structure. Higher glucose and higher BMI were associated with a higher predicted severe-risk probability, but the subtype centers were not arranged along a single linear axis. S3 occupied the high glucose and high BMI region, supporting its interpretation as a gluco-lipotoxic phenotype, whereas S4 showed high severe-risk probability through a different pathway dominated more by blood-pressure and vascular-related signals. Figure 5C further confirms that latent subtypes are nested within, but not reducible to, conventional health classes.

Program-level profiling in Figure 5D summarizes subtype deviations across broader biomedical domains. S3 showed strong glycaemic, lipid, and adiposity shifts; S4 showed the most prominent blood-pressure and vascular-related shifts; S1 showed a relatively stronger hepatic/inflammatory direction; and S5 showed age-related and vascular features. The prototype-risk density in Figure 5E further shows that risk increased along the prototype-distance axis, especially for S3 and S4. This result supports prototype distance as a useful representation of subtype-level risk burden, although it should be interpreted as a model-derived risk marker rather than a causal index.

The subtype cards in Figure 5F provide practical summaries of central clinical values for each subtype. These cards are useful because they translate the latent structure into ordinary occupational health variables such as age, BMI, systolic blood pressure, glucose, ALT, and homocysteine. The abnormality-burden map in Figure 5G provides a second, threshold-based view. It shows that S3 carried a high burden of obesity, hyperglycaemia, high HbA1c, and dyslipidaemia, whereas S4 was more strongly linked to hypertension and vascular-risk abnormalities. S0 had the lowest overall abnormality burden, which is consistent with the resilient-healthy interpretation.

The dynamic and risk-ranking analyses further support subtype prioritization. Figure 5H shows that the subtype composition changed across examination years, again indicating that occupational health risk structure is not static. Figure 5I ranks the subtypes by bootstrapped mean Class III probability and shows that S4 had the highest severe-risk probability (mean approximately 0.91), followed by S3 (approximately 0.57), whereas S0 and S2 had very low severe-risk probabilities. These findings suggest that S4 and S3 should be prioritized for closer review, while S2 may represent a large early-intervention population with milder metabolic deviation.

### 3.6 Practical implications for structure-aware occupational health management

The hidden subtype structure suggests that occupational health examination data may support a more differentiated form of risk interpretation than conventional health-status categories alone. Standard health conclusions remain essential because they are administratively standardized and clinically familiar. However, the subtype analyses in Figures 4, 5 show that workers within the same conventional category may differ in their dominant abnormality patterns, including glycaemic-lipid burden, blood-pressure and homocysteine burden, hepatic/inflammatory deviation, uric-metabolic deviation, and ageing-vascular features (21, 22). Therefore, the proposed framework should be viewed as an interpretive layer that enriches routine classification rather than a replacement for existing occupational health grading.

At the individual level, the subtype framework may help identify different monitoring priorities. For example, workers aligned with the gluco-lipotoxic subtype may require closer review of glucose, adiposity, HbA1c, and lipid-related abnormalities, whereas workers aligned with the hypertensive-hcy subtype may require closer attention to blood pressure and vascular-risk-related indicators (23, 24). The hepato-inflammatory and uric-metabolic subtypes may represent different lower-to-intermediate burden profiles that are not captured by a single overall severity score. These interpretations should remain descriptive and should not be treated as causal disease mechanisms or definitive clinical diagnoses (25).

At the population level, subtype distributions may provide a useful supplement to annual abnormality rates. The temporal and unit-related patterns shown in Figures 2I, 4D,I, 5H indicate that the composition of hidden health burden can shift across examination years and occupational units. Such shifts may reflect changes in cohort composition, work context, examination timing, or other unmeasured factors. Although the present study cannot separate these sources, monitoring subtype composition may help occupational health teams detect structural changes in workforce health burden that are not obvious from isolated biomarker thresholds.

Overall, the practical value of Graph-DAPTN lies not in assigning a more complex label, but in providing a structured map of hidden occupational health heterogeneity. This map can support more targeted follow-up, more nuanced population reporting, and more hypothesis-driven occupational health management, while preserving the central role of conventional clinical judgment.

### 3.7 Limitations

Several limitations should be considered when interpreting these findings. First, although the cohort was large and covered multiple examination years and occupational units, it was derived from a single occupational health system. This may introduce selection bias related to the workforce composition, examination protocol, regional context, and organizational structure.

Second, the study should be interpreted as a repeated cross-sectional analysis rather than a formal longitudinal tracking study, because worker identifiers were not consistently traceable across all years. Therefore, the temporal drift patterns should be understood as population-level changes in subtype composition, not as direct evidence of individual transitions between subtypes. Future studies with stable individual identifiers and follow-up outcomes will be needed to evaluate subtype progression, prognosis, and response to intervention.

Third, missingness and measurement heterogeneity are unavoidable in real-world occupational health data. Although the model incorporated missingness-aware features and preprocessing procedures, residual bias from nonuniform data collection across years, units, and examination workflows cannot be fully excluded. In addition, the subtype labels are model-informed latent phenotypes rather than established clinical disease entities. They summarize reproducible biomarker patterns but do not prove causal biological mechanisms.

Fourth, although Graph-DAPTN improves the interpretability of latent subtype discovery by using grouped biomarker encoding, graph-enhanced representation learning, prototype structure, and subtype-level profiling, the model remains more complex than conventional statistical tools.

## 4 Conclusion

This study builds a graph-enhanced deep representation learning framework for occupational health risk identification based on large-scale real-world data from occupational health examinations. The results show that there are multiple hidden health subtypes with clear biomedical indications under the traditional health classification. These subtypes have different abnormal spectrums in glucose and lipid metabolism, blood pressure, liver burden, uric acid/kidney function, vascular inflammation and age-related risks. Graph-DAPTN not only improves the representation ability of complex occupational health structures, but more importantly, helps occupational health workers see the internal heterogeneity that traditional classification systems cannot fully reveal. Therefore, in future occupational health work, while retaining the traditional hierarchical management framework, data-driven identification of hidden subtypes can be introduced to support more detailed and targeted risk screening and population intervention.

## Data Availability

The original contributions presented in the study are included in the article/Supplementary material, further inquiries can be directed to the corresponding authors.
