# Supplementary material for: Identification of hidden subtypes in occupational health examinations and their biomedical characteristics using graph-enhanced deep representation learning
Source: Front Public Health. 2026 Jul 13;14:1847173. doi: 10.3389/fpubh.2026.1847173 (PMC13402512; doi:10.3389/fpubh.2026.1847173)
Supplement: Supplementary file 2 [file Data_Sheet_2.pdf]

Identification of Hidden Subtypes in Occupational Health Examinations and Their Biomedical Characteristics Using Graph-Enhanced Deep Representation Learning

Zhaoli Wang[1], Xuhui Chen[1], Wei Xiang[2], Ying Wang[2], Yang Sun[3], Yan Pan[4], Chunhuo Zhang[5,*], Yu Ye[2,*]

1 Department of Labor and Health, China Railway Harbin Group Co., Ltd., Harbin 150000, Heilongjiang, China;

2 Harbin Railway Disease Prevention and Control Center, China Railway Harbin Group Co., Ltd., Harbin 150000, Heilongjiang, China;

3 Heilongjiang Provincial Geriatric Hospital, Harbin 150000, Heilongjiang, China

4 School of Medicine, University of Electronic Science and Technology of China, Chengdu 610054, China

5 Heilongjiang Provincial Health Commission, Harbin 150000, Heilongjiang, China

Corresponding author: Huochun Zhang (3031359@qq.com), Yu Ye (yeyuxhb2004@163.com)

Supplementary Appendix A

Conventional Algorithm Comparison and Prediction Benchmark

Methods

We benchmarked conventional supervised classifiers under leave-one-year-out and leave-one-unit-out splits. The compared methods included boosting models, tree ensembles, single decision trees, linear models, distance-based classifiers, probabilistic classifiers, and multilayer perceptrons. Macro-F1 and balanced accuracy were used as the primary evaluation metrics because the occupational health classes were imbalanced.

Graph-DAPTN was evaluated separately under repeated cross-domain holdouts. These analyses tested whether the graph-enhanced representation remained predictive under temporal and unit-level domain shifts. In the revised main manuscript, subtype-specific validation is reported separately because hidden subtype discovery and conventional health-status prediction address different scientific questions.

Results

Conventional boosted and tree-based models achieved high health-status prediction performance. HistGradientBoosting reached macro-F1 values of 0.963 in unit-out validation and 0.961 in year-out validation. GradientBoosting, RandomForest, and DecisionTree also performed strongly. These results indicate that conventional health-status labels are predictable from occupational examination variables, but they do not directly answer whether hidden subtype structure exists beneath those labels.

Graph-DAPTN showed robust but not uniformly superior supervised prediction performance across cross-domain internal holdouts. Mean macro-F1 across unit holdouts ranged from 0.806 to 0.893, and across year holdouts from 0.782 to 0.887. This pattern supports cross-domain robustness of the learned representation, while also reinforcing the revised framing that the main contribution of Graph-DAPTN lies in subtype-oriented representation learning rather than simple prediction superiority.

Supplementary Figure S1

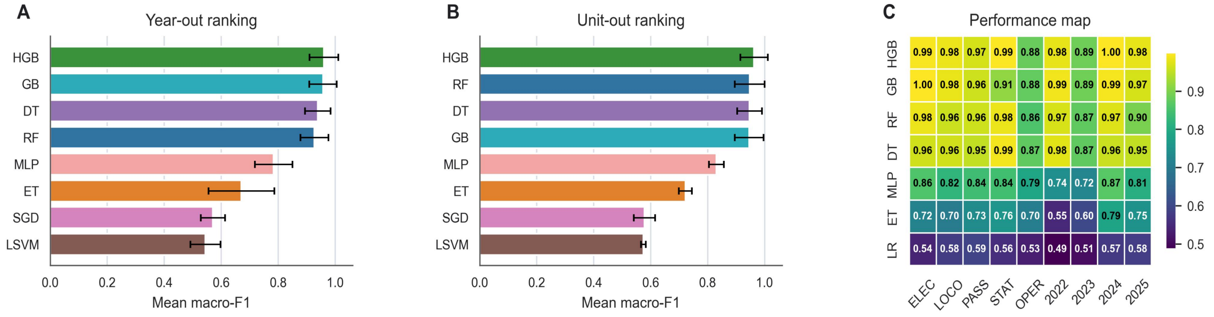

Supplementary Figure S1, panels A-C.

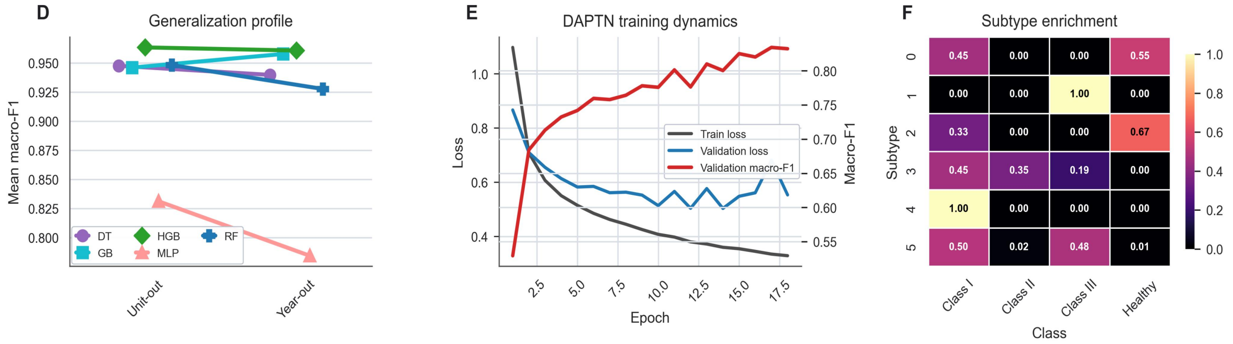

Supplementary Figure S1, panels D-F.

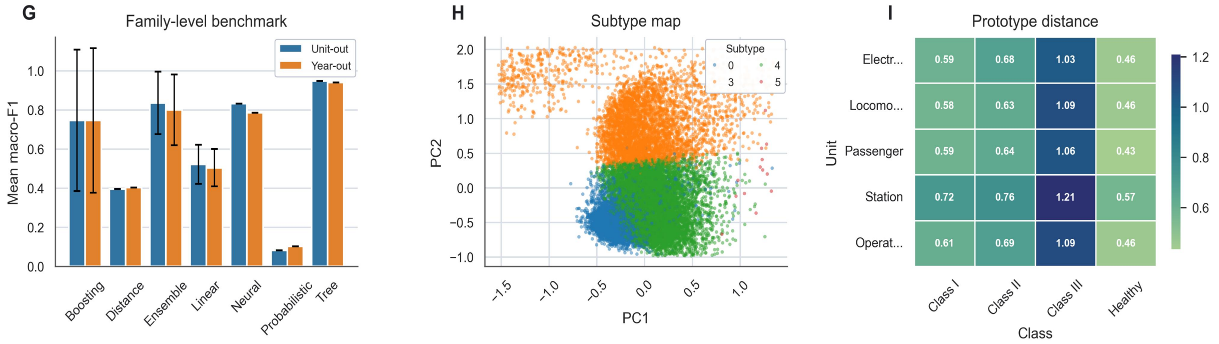

Supplementary Figure S1, panels G-I.

Supplementary Figure S1. Conventional health-status prediction benchmark. Panels A-B rank conventional supervised models under leave-one-year-out and leave-one-unit-out validation. Panel C summarizes performance across units and years. Panels D-G show model-family and generalization profiles. Panel E shows DAPTN training dynamics. Panel F shows subtype enrichment across conventional classes. Panel H shows the subtype map, and panel I summarizes prototype-distance patterns across units and classes.
